# Ecology of Root Colonizing *Massilia* (Oxalobacteraceae)

**DOI:** 10.1371/journal.pone.0040117

**Published:** 2012-07-11

**Authors:** Maya Ofek, Yitzhak Hadar, Dror Minz

**Affiliations:** 1 Institute of Soil, Water and Environmental Sciences, Agricultural Research Organization, Bet Dagan, Israel; 2 The Robert H. Smith Faculty of Agriculture, Food and Environment, The Hebrew University of Jerusalem, Rehovot, Israel; Virginia Tech, United States of America

## Abstract

**Background:**

Ecologically meaningful classification of bacterial populations is essential for understanding the structure and function of bacterial communities. As in soils, the ecological strategy of the majority of root-colonizing bacteria is mostly unknown. Among those are *Massilia* (Oxalobacteraceae), a major group of rhizosphere and root colonizing bacteria of many plant species.

**Methodology/Principal Findings:**

The ecology of *Massilia* was explored in cucumber root and seed, and compared to that of *Agrobacterium* population, using culture-independent tools, including DNA-based pyrosequencing, fluorescence *in situ* hybridization and quantitative real-time PCR. Seed- and root-colonizing *Massilia* were primarily affiliated with other members of the genus described in soil and rhizosphere. *Massilia* colonized and proliferated on the seed coat, radicle, roots, and also on hyphae of phytopathogenic *Pythium aphanidermatum* infecting seeds. High variation in *Massilia* abundance was found in relation to plant developmental stage, along with sensitivity to plant growth medium modification (amendment with organic matter) and potential competitors. *Massilia* absolute abundance and relative abundance (dominance) were positively related, and peaked (up to 85%) at early stages of succession of the root microbiome. In comparison, variation in abundance of *Agrobacterium* was moderate and their dominance increased at later stages of succession.

**Conclusions:**

In accordance with contemporary models for microbial ecology classification, copiotrophic and competition-sensitive root colonization by *Massilia* is suggested. These bacteria exploit, in a transient way, a window of opportunity within the succession of communities within this niche.

## Introduction

Within the soil, the plant rhizosphere is nothing short of a gold mine in terms of carbon availability for its microbiota. Soil bacteria maintain a large number of adaptive traits which enable them to prosper in this highly competitive niche [1]. Those traits have been studied extensively for some bacterial species, including pathogens [2], [3], mutual symbionts [4] and a few commensal, plant-growth-promoting species [1], [5].

Technical advances in recent years have enabled comprehensive cultivation-independent studies of the composition of root and rhizosphere bacterial communities [6]. As a result of such studies, the number of bacterial species and genera classified as root-associated continues to rise [6], [7]. For most of these ‘novel’ root-associates, their ecology and life strategies are unknown. Among those are *Massilia* (Oxalobacteraceae), a group of bacteria of an emerging interest in recent years, described in a broad range of niches.

Members of the genus *Massilia* were first isolated from clinical samples [8], [9] and were characterized as aerobic, flagellated, non-spore forming rods [8], [10]. *Massilia* were thereafter isolated and detected in environmental samples of many sources, including air, aerosols and dust samples [11]–[13], freshwater [14], soils [15], [16] and phyllosphere [17], [18]. In recent years, *Massilia* had been detected in- and isolated from the rhizosphere and roots of many plant species [19]–[29]. Although this group of bacteria appears to be an important component of the rhizosphere, *Massilia* and Oxalobacteraceae in general were seldom specifically examined in rhizosphere studies [19], [20]. Therefore, association of *Massilia* with roots and different root compartments as well as population dynamics in the root niche is little known.

In previous studies, we have reported that indigenous *Massilia* (Oxalobacteraceae) populations can achieve high dominance in the cucumber rhizosphere and spermosphere, but are also sensitive to conditions in the growth medium [30], [31]. Here, a qualitative and quantitative investigation of *Massilia* colonization of cucumber seeds and roots was performed in order to gain further understanding of their association with different rhizosphere and spermosphere components, and their dynamics in this environment.

## Results

### Composition of Seed and Root Colonizing *Massilia* and Other Oxalobacteraceae

**Figure 1:**
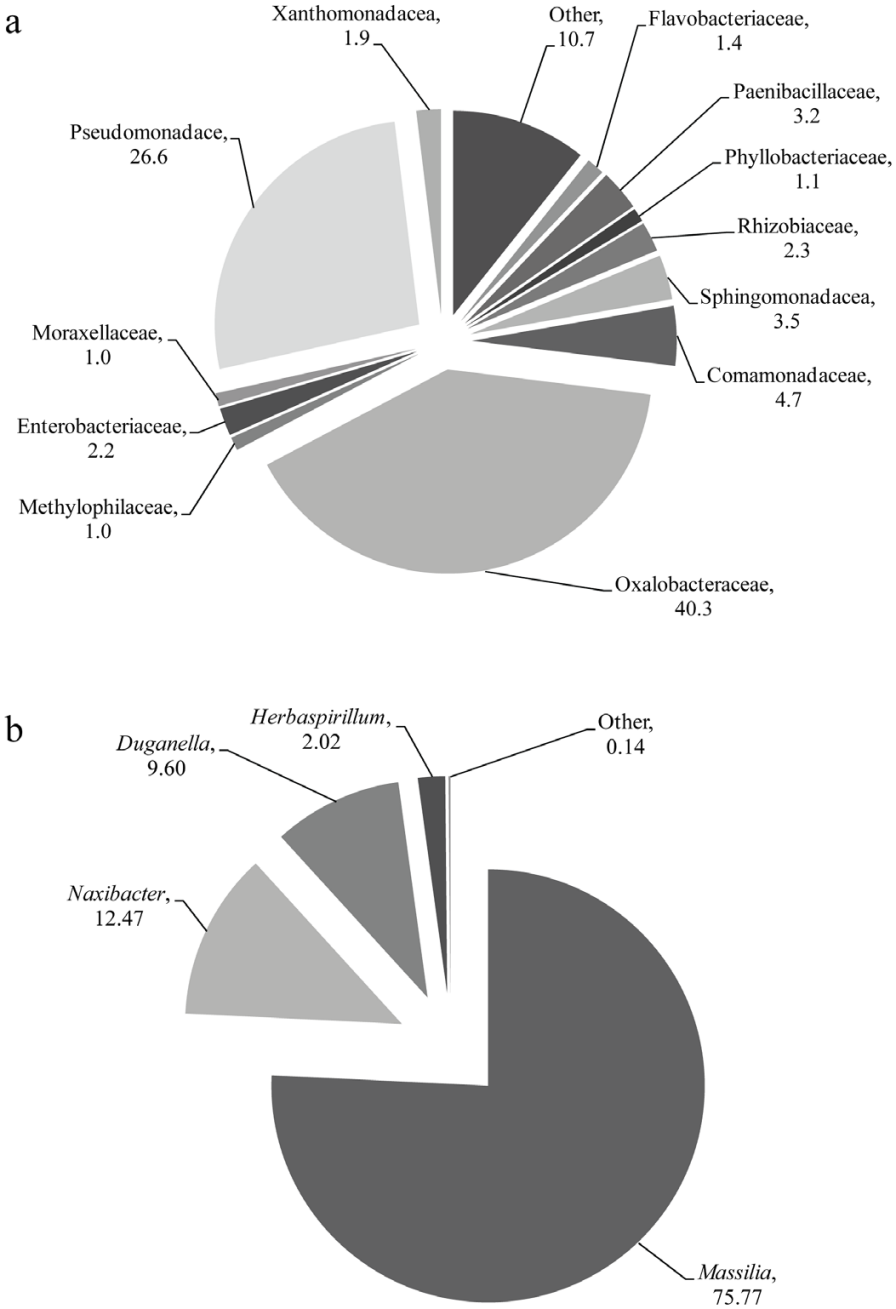
Composition of cucumber (*Cucumis sativus*) seed- and root-colonizing bacterial (a) and Oxalobacteraceae community (b), based on 454-pyrosequencing of general bacterial 16S rRNA gene fragments. Sequences were obtained from samples of seeds (1 day) and roots (2, 7 and 21 days old) grown in perlite and compost-amended perlite, with and without inoculation with *Pythium aphanidermatum* (1day only). Numbers indicate the relative abundance of the indicated taxon (% of total bacteria). Families for which relative abundance was ≥1% are included in panel a.

**Figure 2:**
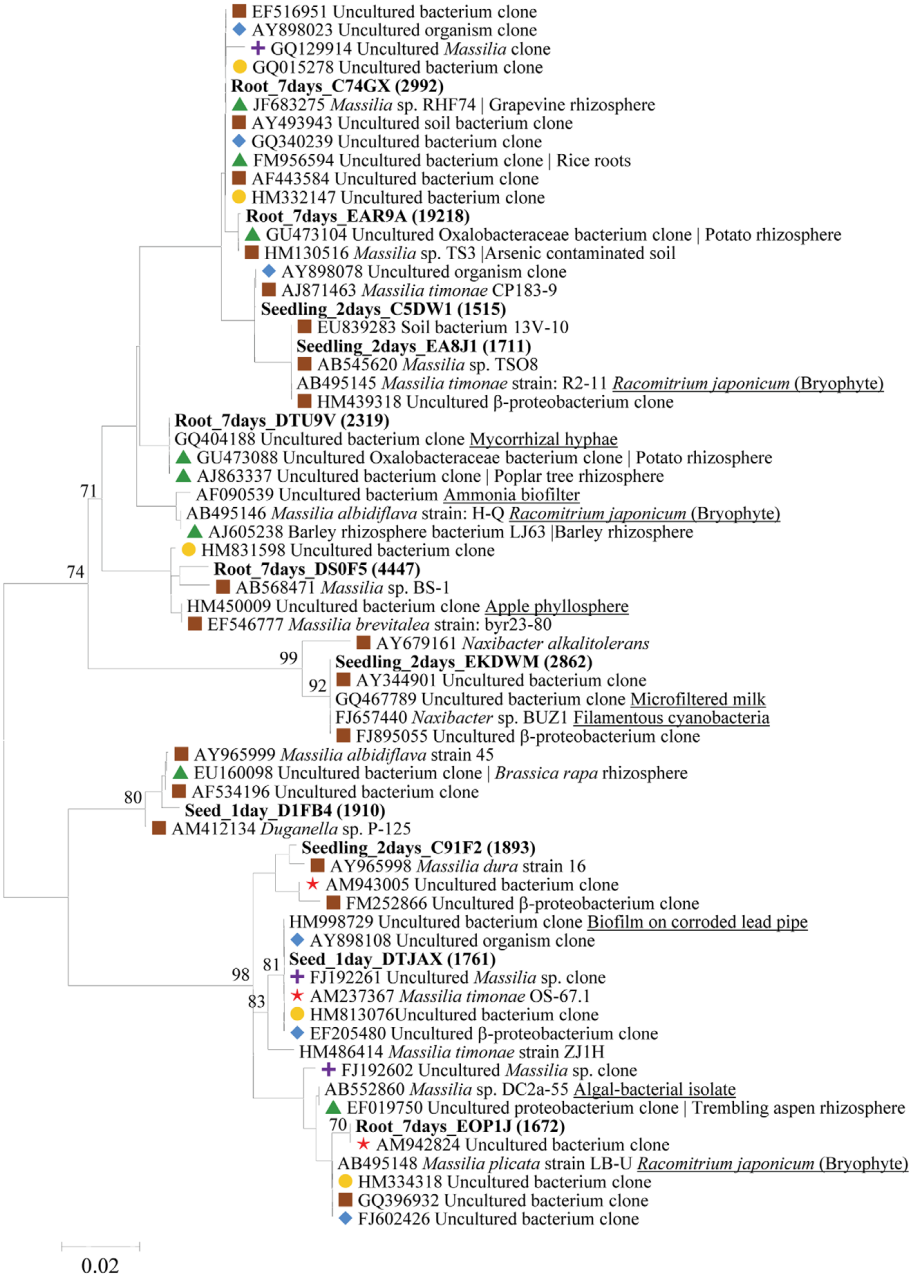
Neighbor-joining tree depicting representatives of the most abundant Oxalobacteraceae OTUs (in bold; number of sequences represented are in brackets) and related sequences from the NCBI database. The tree was calculated using the Kimura two-parameter model. The scale bar represents number of substitutes per site. Numbers at the nodes indicate bootstrap values (1000 replicates). Symbols indicate sequence origin: brown square- soil; green triangle- rhizosphere; yellow circle- human and mouse skin swabs; blue diamond- fresh water; red star- air and dust; and purple cross- clean room. Sequences from other origins are underlined.

The composition of bacteria colonizing cucumber seeds, seedlings and roots was assessed by high-throughput sequencing of 16S rRNA gene fragments, amplified directly from the extracted total DNA. Samples were collected at distinct growth stages: radicle emergence (24 hours), primary root extension (48 hours), first true leaf (7 days) and vine-tip-over (4–5 true leaves, 21 days). Table S1 lists the number of sequences obtained from each of the different treatment and plant ages. Across all samples, a total of 181,163 bacterial sequences were obtained following elimination of suspected chimeras, low-quality sequences and short sequences (<300 bp). Of these, 73,067 sequences (40.3%) were affiliated with the Oxalobacteraceae (Fig. 1a). Four different genera among Oxalobacteraceae were substantially (>1%) represented in the Oxalobacteraceae sequence pool (Fig. 1b). Among these, *Massilia* were by far the most predominant, followed by the genus *Naxibacter*. The eleven most abundant OTUs (<97% sequence similarity between the sequences within each OTU) comprised 58% of Oxalobacteraceae sequences, and are presented along with their closest published relatives in Figure 2. The most abundant *Massilia* sequences were identical or highly similar (99% and above) to sequences previously retrieved from roots and rhizosphere of different plants and soils.

### *Massilia* Colonization of Seeds and Roots

**Figure 3:**
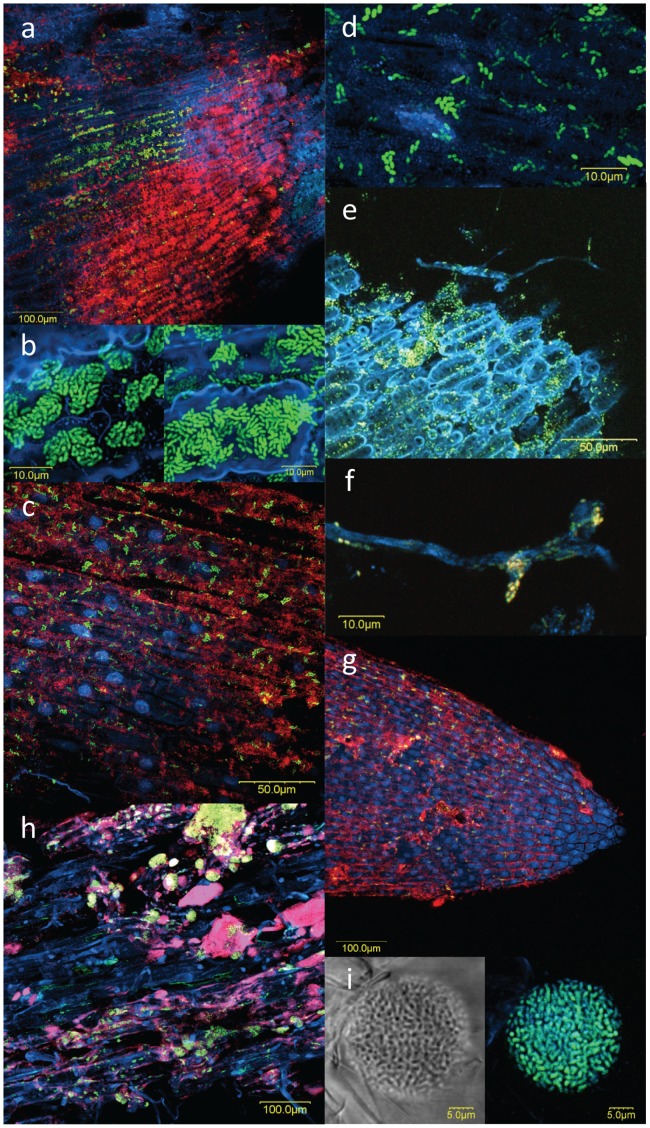
Cucumber seed and root colonization by *Massilia*. FISH-CLSM analyses of the plant samples: blue- DAPI stain; red- total bacteria; green- *Massilia*. a,b) Seed radicle after 24 h in compost-amended perlite; c,d) seed coat after 24 h in compost-amended perlite; e,f) seed coat after 24 h in *Pythium aphanidermatum*-inoculated perlite; g) root tip after 48 h in perlite; h) mature zone of primary root after 48 h in perlite; colony on root after 48 h in perlite: bright-field image on the right, *Massilia* probe and DAPI stain on the left. Arrows indicate *Pythium* hyphae.

FISH-CLSM was used to examine seed and root surface colonization by *Massilia*, using a specifically designed probe (Fig. 3). *Massilia* were undetected on the coat or radicle of seed germinated in perlite for 24 h. In contrast, clusters of few *Massilia* cells, as well as dense *Massilia* clusters were found on the coat of seeds germinated for 24 h in compost-amended perlite (Fig. 3a). Those were found in mixed colonies with other bacteria, but also in exclusive ones, over the surface of the coat cells as well as in grooves between adjacent cells. The dense clusters morphology indicated multiplication taking place (Fig. 3b). By then, *Massilia* small clusters had appeared on the emerging radicle, embedded within films of other bacteria (Fig. 3c,d). On seeds germinated in *Pythium*-infested perlite, *Massilia* made up the vast majority of the colonizing bacteria (Fig. 3e). As on the surface of seeds geminating in compost, *Massilia* were attached to the surface of cells and grooves between cells. In addition, *Massilia* were found physically attached to- and made up the majority of bacteria colonizing the *Pythium* hyphae themselves (Fig. 3e,f). Following 48 h of germination, *Massilia* were rarely detected on primary roots emerging in the compost containing medium (not shown). In contrast, *Massilia* colonized the root tip of roots emerging in the un-amended perlite (Fig. 3g), where they appeared mainly in small clusters. On mature parts of those roots, large mixed as well as exclusive clusters of *Massilia* were formed (Fig. 3h,i). The morphology of many of these clusters suggested active proliferation of *Massilia* in this niche (Fig. 3i).

### Quantitative Real-time PCR Determination of *Massilia* Abundance

The absolute abundance of *Massilia* (*i*.*e*. *Massilia* targets normalized to the plant *tef* gene targets) in seed and root samples were determined by qPCR. Both plant developmental stage and growth medium had significant effects on *Massilia* abundance (*P*<0.001) and showed a significant interaction (*P*<0.001), according to analysis of variance test. Generally, *Massilia* abundance decreased with plant age (Table 1). In untreated perlite, the *Massilia* population dramatically increased from 0.03 to 2624 targets per plant *tef* between the first and second days of growth. By day 7 (first leaf), the population size was reduced to one-tenth and by day 21 (vine-tip-over) it was further reduced by over two orders of magnitude. *Massilia* population size dynamics were affected similarly by the addition of compost, of *Streptomyces* sp. or their combination to the perlite medium (Table 1). Following these treatments, *Massilia* population size climaxed as early as 1 day after seed germination. By day 7, population size was 1.7 to 2.2 orders of magnitude lower in the different treatments as compared to perlite. By 21 days, the effect of *Streptomyces* sp. inoculation on population size was no longer significant, while the effect of compost amendment persisted.

**Table 1:** Quantitative assessment of *Massilia* spp. population size.

			log *Massilia* spp. plant *tef* ^−1^			
			1 day	2 days	7 days	21 days
Medium	*Pythium*	*Streptomyces*				
Perlite	–	–	−1.8 (0.18)^a^ *	3.35 (39.4)	2.19 (55.1)	−0.13 (1.94)
	–	+	3.03 (62.1)^b^	2.78 (22.9)	0.49 (2.50)	−0.11 (1.81)
	+	–	3.49 (85.9)^bc^			
Perlite + compost	–	–	3.15 (15.1)^bc^	2.49 (5.3)	0.21 (1.12)	−0.72 (0.58)
	–	+	3.61 (9.2)^c^	2.44 (2.7)	−0.03 (1.05)	−0.76 (0.56)
	+	–	2.99 (17.8)^b^			
				Critical range** = 0.406 (8.26)		

Cucumber seed- and root-associated *Massilia* spp. and total bacteria targets were quantified using specific qPCR assays and normalized to the plant tef gene. The averages of the absolute and the relative (to general 16S rRNA gene targets, in brackets) abundances are presented (n = 4). The sampling days represent 4 plant developmental stages: Germination (1 day), primary root formation (2 days), first leaf (7 days) and vine-tip over (21 days).

* Different letters indicate significant difference between samples after 1 day of germination. Determined by one-way ANOVA and post-hoc Tukey test (*P*<0.05).

** Determined by factorial ANOVA and post-hoc Newman-Keuls test (For all samples excluding *Pythium* inoculated ones).

Inoculation with the *Pythium* resulted in contrasting effects on the size of *Massilia* population of seeds germinating in perlite compared to compost-amended media (Table 1). While in perlite the seed *Massilia* population increased significantly, compared to the un-inoculated control, in the compost medium *Massilia* population size was unchanged. This contradiction may reflect the effective suppression of *Pythium* development on the seeds due to compost amendment [31].

### Quantitative Real-time PCR Determination of *Agrobacterium tumefacience/radiobacter* Clade Abundance

For comparison to *Massilia* spp. population, we chose to examine the population dynamics of another indigenous root population, which was found to be among the dominant cucumber root populations [30]. Based on mass sequencing data, sequences affiliated (98%–100% sequence similarity) with *Agrobacterium tumefacience*/*radiobacter* (*Agrobacterium* spp.) clade comprised between 0 and 15.7% of the sequences in different samples. Using a specifically designed primer pair, the abundance of this group was determined by real-time qPCR, relative to the plant *tef* gene (absolute abundance), and relative to the total bacteria (Table 2). Similar to the results for *Massilia*, the absolute abundance of *Agrobacterium* spp. was significantly affected by the plant growth medium and plant age (*P*<0.01) and showed a general trend of decline with plant age. However, degree of variation in absolute abundance between samples was markedly diminished. For example, in perlite, *Agrobacterium* spp. abundance had increased by 2.4 orders of magnitude between 1 and 2 days of plant growth, which was ∼0.2% of the clime in *Massilia* abundance under the same conditions (Tables 1 and 2). In each of the different treatments, the average minimum to maximum difference in population size was 2.29±0.67 orders of magnitude for *Agrobacterium* spp. compared to 4.13±0.85 orders of magnitude for *Massilia* spp.

**Table 2:** Quantitative assessment of *Agrobacterium* spp. population size.

			log *Agrobacterium* spp. plant t*ef* ^−1^			
			1 day	2 days	7 days	21 days
Medium	*Pythium*	*Streptomyces*				
Perlite	–	–	−1.58 (0.26)^a^ *	0.56(0.11)	1.34 (7.49)	0.53 (8.59)
	–	+	0.82 (0.63)^b^	2.04 (4.15)	0.68 (4.03)	0.63 (10.28)
	+	–	1.53 (1.1)^c^			
Perlite + Compost	–	–	2.39 (2.62)^d^	1.67(1.20)	1.06 (7.00)	0.28 (5.55)
	–	+	3.12 (3.03)^e^	1.76(0.65)	1.37 (13.17)	0.36 (7.45)
	+	–	2.3 (3.86)^d^			
				Critical range** = 0.312 (2.18)		

Cucumber seed- and root-associated *Agrobacterium* spp. and total bacteria targets were quantified using specific qPCR assays and normalized to the plant *tef* gene. The averages of the absolute and the relative (in brackets) abundances are presented (n = 4). The sampling days represent 4 plant developmental stages: Germination (1 day), primary root formation (2 days), first leaf (7 days) and vine-tip over (21 days).

* Different letters indicate significant difference between samples after 1 day of germination. Determined by one-way ANOVA and post-hoc Tukey test (*P*<0.05).

** Determined by factorial ANOVA and post-hoc Newman-Keuls test (For all samples excluding *Pythium* inoculated ones).

### Correspondence between Absolute Abundance and Relative Abundance in *Massilia* spp. and *Agrobacterium* spp

**Figure 4:**
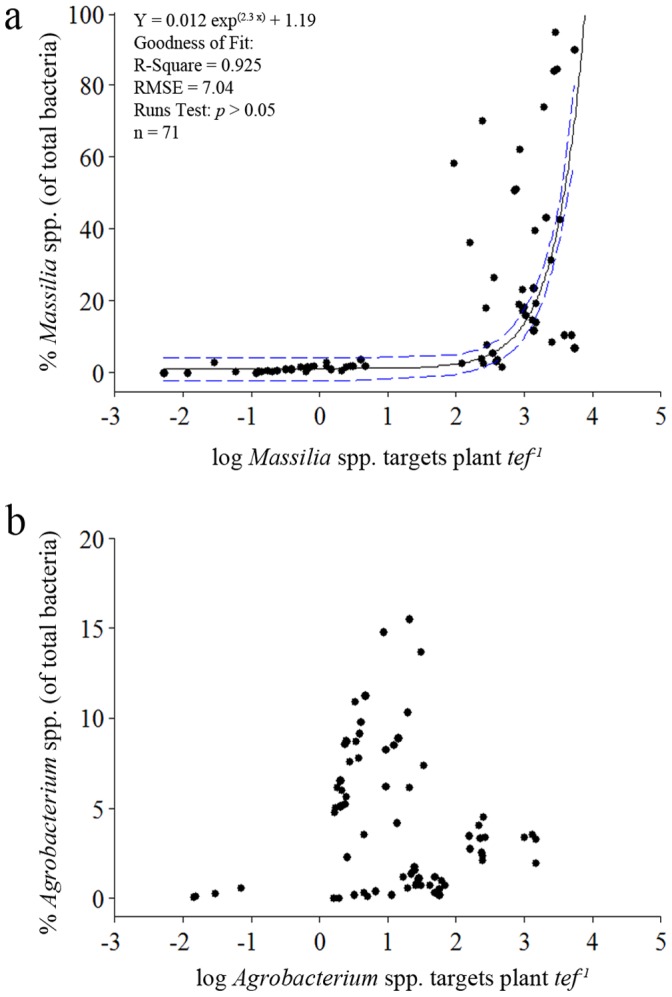
Best-fit analysis of the interaction between relative and absolute abundance of seed- and root-colonizing *Massilia* (a) and *Agrobacterium radiobacter* (b). Relative and absolute abundances, normalized to the plant *tef* gene, were determined by qPCR assay. *df*: degrees of freedom; MSE: mean square error.

Relative abundance of *Massilia* spp. and *Agrobacterium* spp. (both normalized to total bacteria) also varied greatly between the different treatments and plant developmental stages (Tables 1 and 2). When *Massilia* relative abundance was plotted against the absolute abundance (expressed as log *Massilia* targets *tef* ^−1^), samples could be divided into two groups (Fig. 4a): the first group (n = 32) included samples with absolute abundance below 0.7 log targets *tef* ^−1^ and relative abundance values ranging between 0.04% and 3.64% with a mean of 1.25 log targets *tef* ^−1^. The second group (n = 39) included samples with *Massilia* absolute abundance values above 1.9 log targets *tef* ^−1^ and with highly variable relative abundance values, ranging between 1.86% and 94.76% with a mean of 30.94 log targets *tef* ^−1^. We have further examined the relatedness between these two variables by non-linear modeling. The robust non-linear regression analysis approach was used to evaluate exponential model of correspondence. For a three-parameter model [Y = a·exp^(b·X)^+c], the parameter estimates and goodness of fit statistics revealed a clear positive exponential correspondence between *Massilia* spp. absolute abundance and relative abundance (Fig. 4a). The parameter estimates and 95% confidence intervals were: a = 0.012 (−0.007–0.031); b = 2.32 (1.875–2.757); c = 1.19 (−1.12–3.5). A Runs Test performed did not find systematic deviation between the model curve and the data points (*P*>0.05), further supporting the goodness of fit of the model. In the case of the *Agrobacterium* spp. population, the highest relative abundance values were achieved at the later developmental stages examined (Table 2). However, there was no evidence for a trend relating the absolute and relative abundance values for this group (Fig. 4b). In accordance, all attempts for modeling resulted in failure of convergence, even when using robust non-linear regression method.

### Response of *Massilia* to Soil Conditions

**Figure 5:**
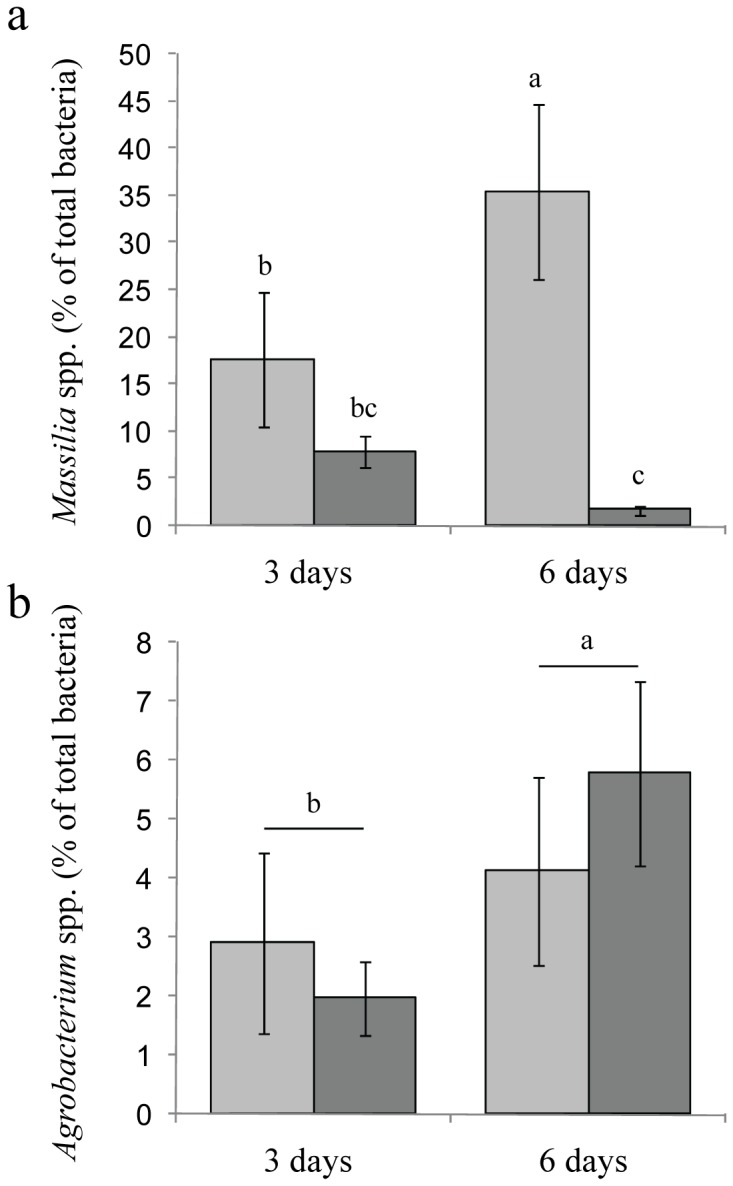
Relative abundance of *Massilia* (a) and *Agrobacterium* spp. (b) on cucumber seedling roots as determined by real-time quantitative PCR. Cucumber seeds were germinated and grown under greenhouse conditions in sandy soil for 6 days and then transplanted into the same soil (bright grey) or wild rocket amended soil (dark grey). Roots were sampled 3 and 6 days after transplantation and DNA extracted from the samples was used for quantification of total bacteria, *Massilia* spp. and *Agrobacterium* spp. Means and standard deviations are presented (n = 5). Different letters indicate significant differences between the means according to factorial ANOVA (*P*<0.05) followed by the post-hoc Tukey HSD test.

The sensitivity of *Massilia* to organic amendment was further examined in an agricultural soil, under greenhouse conditions. The organic amendment consisted of dry residues of wild rocket (WR, *Diplotaxis tenuifolia*), which resulted (similar to the compost amendment) in evolution of soil suppressiveness towards plant pathogens [32]. Six-days old cucumber seedlings (grown in un-amended soil) were transplanted into either un-amended soil or soil amended with WR residues, and harvested 3 and 6 days after transplantation. The relative abundance of *Massilia* spp. and *Agrobacterium* spp. colonizing the roots was determined by real-time qPCR. Three days after transplantation, the relative abundance of *Massilia* on roots was similar in un-amended and WR amended soil treatments (Fig. 5a). Six days after transplantation, the relative abundance of *Massilia* had doubled in the un-amended soil but had dropped by 78% in the WR amended soil (P<0.05). In contrast, soil amendment with WR had no significant effect on *Agrobacterium* spp. relative abundance 3 or 6 days after transplantation of the seedlings (Fig. 5b). Nevertheless, relative abundance of *Agrobacterium* spp. had significantly increased between 3 and 6 days (*P*<0.05).

## Discussion

The genus *Massilia* was defined recently [8] and since then, its members were described in highly diverse environments. In recent years, reports of *Massilia* as rhizosphere and endorhizal colonizers have become frequent [19]–[31], [33]. In addition, some isolates of *Massilia* exhibited *in vitro* attributes related to plant growth promotion, including IAA production [29], siderophore production [23] and *in vitro* antagonism towards *Phytophthora infestans* [24]. Acknowledging its broad occurrence and importance, we endeavored to learn more about the below ground ecology of plant-*Massilia* association.

*Massilia* comprised over 75% of cucumber seed- and root-associated Oxalobacteraceae (Fig. 1) and were found in all of the extremely different conditions examined here. The phylogenetic analysis revealed that the dominant *Massilia* spp. found here clearly belongs to a soil-associated group (Fig. 2). Some representatives, including of the most dominant OTU, were also highly related to *Massilia* spp. from grapevine [34], potato [35], poplar tree [36] and other plant species rhizosphere or endorhiza (Fig. 2). At the same time, close relatives were also retrieved from nutrient-poor fresh water, hyper-arid environments, including air, dust, and even clean rooms (Fig. 2), and from highly heavy metal contaminated sites [22], [29], [37], [38]. Therefore, tolerance towards a-biotic (non-resource) stresses may be common among members of this genus.

Effective attachment is thought to be a basic requirement for successful root colonization [39]. *Massilia* efficiently colonized all surfaces examined here, *i.e*. seed coat and radicles, roots and even the hyphae of *Pythium* infecting germinating seeds (Fig. 3). Previously, it was shown that *Massilia* and other closely related Oxalobacteraceae can rapidly colonize hyphae of mycorrhizal fungi [40]. In addition, *Massilia* isolates were obtained from marine filamentous Cyanobacteria [41] and from freshwater filamentous algae and Cyanobacteria (Fig. 2). Furthermore, *Massilia*-related sequences have been detected on human and mouse skin [42], [43]. Hence, efficient attachment to biological surfaces may be a common trait in members of this genus.

The almost exclusive colonization of the *Pythium* hyphae by *Massilia* calls for attention to this specific interaction. Bacterial colonization of the hyphae of Oomyces and fungi, including mycorrhiza, is a common phenomenon [44]. Many of those bacteria feed on living hyphal cells and consume fungal exudates, which may be rich in sugars and amino acids. In some cases, the effect of the interaction on fungal performance may be positive, as in the example of mycorrhizal helper bacteria [44]–[45]. However, the interaction may have inhibitory or destructive results, which in the context of plant-pathogen interaction may lead to pathogen control [3]. Functional traits associated with bacterial mechanisms of phytopathogen control were identified in different *Massilia* isolates, including production of siderophores [23] and extracellular lytic enzymes [26], [45]–[46]. In addition, several *Massilia* isolates can degrade cellulose [23], the principle component of *Pythium* cell-wall. However, reports of *in vitro* antagonism are rare [24], [47] and no report of *in vivo* biocontrol was found. The data provided in this study cannot support a *Pythium*-*Massilia* interaction beyond commensalism.

Contemporary studies have attempted to lay a framework for ecologically meaningful classification of soil bacteria [48]–[49]. Along the copiotrophic-oligotrophic life strategy continuum, copiotrophs are considered to have higher growth rates compared to oligotrophs, but also high sensitivity to nutrient availability and competition, due to higher maintenance requirements as well as lower substrate affinity. The applicability of this principle was demonstrated by Fierer *et al*. (2007) at phyla- and class-level of taxonomic resolution. Based on these characteristics, it is expected that for copiotrophic populations, population size (absolute abundance) and dominance (relative abundance) will be positively linked. Here, we found that *Massilia* spp. absolute (log based) and relative abundances were positively linked (Fig. 4a). In addition, using a robust non-linear regression method, an exponential growth model could be determined for the interaction between these two variables. Therefore, *Massilia* spp. behavior at the root niche may be classified as copiotrophic. Indeed, the quantitative real-time PCR results (Table 1) as well as the *in situ* evidence (Fig. 3g,h) supported high growth rate of plant surfaces-associated *Massilia* population.

Plant associated *Massilia* spp. have outgrown *Agrobacterium* spp. in untreated perlite medium by almost three orders of magnitude between the first and second days of seed germination (Tables 1 and 2), even though the initial abundances were similar. In addition, variation in population size and dominance, related to plant developmental stage or growth medium were much larger for *Massilia* spp. compared with *Agrobacterium* spp. (Tables 1 and 2, Fig. 5). All these evidence are in line with the previously proposed general model of copiotrophs classification [48].

Copiotrophic behavior or *r*-selection ecology characterizes populations involved in early stages of microbial succession, while more oligotrophic populations are positively selected in later stages and their relative abundance increases [48]–[51]. In accordance, the age-linked dynamics found here relate *Massilia* to early stages of succession of root system microbiome development, while the *Agrobacterium* spp. population was related to later stages of succession. Involvement of Oxalobacteraceae and particularly of *Massilia* in early stages of bacterial succession was previously reported in soil [50] and soil amended with fresh plant residues [52], [53]. Within soils, the rhizosphere niche is unique as the growing roots provide constant supply of labile organic carbon. Thus, carbon availability is not considered a limiting factor in this niche [54]. Therefore, while loss of dominance of *Massilia* could be attributed to reduction in quantity and quality of available organic carbon in soils [50], on the root itself, such loss of dominance may be better explained by interspecies competition for other resources. Such sensitivity to competition was indicated for *Massilia* colonizing the rhizosphere soil of *Salix viminalis* [23]. Here, competitive conditions were promoted by amendment of the perlite with compost, or soil amendment with wild rocket residues. Both these amendments resulted in suppressiveness towards plant pathogens [31]–[32] which is often correlated with heightened nutrient competition among soil inhabitants [3], [55]–[56]. In both cases, sensitivity of *Massilia* was demonstrated. Additionally, the response of *Massilia* to the introduction of potential competitor (by inoculation with *Streptomyces* sp.) was also significant though transient. The sensitivity to biotic stress stands in contrast to the apparent tolerance to a-biotic stressors, including drought and heavy metals, related to members of this genus [11], [16], [22]. A-biotic (non-resource) stress tolerance is not particularly attributed to copiotrophic organisms. Nevertheless, mechanisms of stress tolerance are diverse and do not necessarily contradict copiotrophic life style. Evidently, much is yet to be studied regarding the life strategy of *Massilia*.

In conclusion, seed- and root-colonizing *Massilia* belong to a bacterial group that is found in highly diverse environments, but are primarily soil bacteria. In the rhizosphere ecology they function as copiotrophs. They appear to exploit a window of opportunity in early succession of the root niche, when sufficient carbon and energy sources are present, but before competition with other rhizosphere microorganisms becomes limiting. During this short period, they attach to plant surfaces and achieve extremely high dominance due to high proliferation rates.

## Materials and Methods

### Growth Media Preparation and Plant Growth Conditions

Perlite particles larger than 2 mm in diameter were collected by sieving, washed with tap water and air-dried. Biosolids compost, suppressive toward several plant pathogens [57], [58], was sampled from a commercial composting facility (Shacham, Givaat Ada, Dlila Facility, Israel). The compost was washed with two volumes of tap water and air-dried. The dry compost was mixed with perlite at a ratio of 1∶4 (v/v). The compost-amended as well as untreated perlite media were hydrated with half-strength Hoagland solution [59]. When indicated, *Pythium aphanidermatum* mycelial homogenate (200 mL L^−1^) or *Streptomyces* sp. S1 spores (10^6^ L^−1^) were added to the dry material prior to hydration as previously described [30]. *Pythium* inoculum density was determined in a calibration experiment to produce approximately 70 to 85% disease incidence in the untreated perlite control.

Cucumber seeds (*Cucumis sativus* cv. ‘Kfir’, Zeraim, Gedera, Israel) were surface-sterilized by soaking in 3% sodium hypochlorite for 1.5 min followed by 70% ethanol for 1.5 min and washing three times with sterile water.

### Preparation of *P. aphanidermatum* Mycelium Homogenate and *Streptomyces* sp. S1 Spores

*Pythium aphanidermatum* (isolate 64) was kindly provided by Prof. Y. Ben-Yefet, Department of Plant Pathology, The Volcani Center’s Institute of Plant Protection, Bet Dagan, Israel. *Pythium* cultures were maintained on potato dextrose agar medium. For preparation of inocula, mycelia were grown in liquid V8-cholesterol medium (10% V8 juice, 0.1% CaCO_3_, 0.015% cholesterol) at 30°C for 4 days. Immediately prior to the inoculation process, mycelia from 200 mL V8-cholesterol medium were harvested, washed twice in sterile distilled water and homogenized in 100 mL of half-strength Hoagland nutrient solution.

*Streptomyces* sp. S1, able to antagonize *P. aphanidermatum in vitro*, was isolated from the disease-suppressive biosolids compost, and was shown to elicit a substantial shift in bacterial community composition in young cucumber seedlings, including *Massilia* and *Agrobacterium* populations [30]. Preparation of appropriate inocula was as previously described [30]. Briefly, *Streptomyces* sp. S1 cultures were grown on mannitol-soy agar for 14 days and spores were scraped from the surface and washed in sterile saline solution (0.85% NaCl). Spore density was determined by dilution plating on yeast-extract malt-extract agar and adjusted to 10^9^ spores mL^−1^.

### Experimental Design, Sampling Procedure and DNA Extraction

The experiment consisted of six different plant growth media (PGM): untreated perlite, compost-amended perlite, *Streptomyces* sp. S1 (*S1*) -amended perlite, compost and *S1* amended perlite, *Pythium*-inoculated perlite, and *Pythium*-inoculated compost-amended perlite. Plant material was sampled at four different time points: 1, 2, 7 and 21 days. These time points represented distinct developmental stages of the cucumber plant: radicle emergence, primary root extension, first true leaf and vine tip over (4–5 true leaves) respectively. The *Pythium*-inoculated treatments were sampled only on the first day of germination. For the 1- and 2-day time points, seeds were sown in PGM packed Petri dishes (12 seeds per plate) in four replicates (each replicate consisted of five Petri dishes). Plates were incubated at 30°C with constant illumination. For the 7- and 21-day time points, seeds were sown in 200-mL plastic pots packed with PGM (4 seeds per pot) in four replicates and then grown under greenhouse conditions at 30°C with constant illumination.

Seeds or roots were collected from the PGM, placed in 50-mL plastic tubes and washed twice with sterile saline solution. From each replicate, 12 seeds or 250 mg of roots were transferred to DNA-extraction tubes for further processing. Extraction of DNA was carried out using the PowerSoil® DNA Isolation Kit (Mo Bio Laboratories, Inc., California, USA) according to the manufacturer’s instructions. DNA concentrations were determined using the NanoDrop ND1000 spectrophotometer (NanoDrop Technologies, Wilmington, USA). From samples grown for 1 and 2 days, 24 seeds were fixed in 15 mL FAA (5% formaldehyde, 5% acetic acid, in ethanol) for 24 hours at room temperature and then stored at −20°C.

### Sequencing of 16S rRNA Gene Fragments

DNA extracted from seed and root samples collected from untreated perlite, compost-amended perlite, *Pythium*-inoculated perlite and *Pythium*-inoculated compost-amended perlite was subjected to mass sequencing of fragments of the small subunit rRNA. 16S rDNA bacterial tag-encoded FLX amplicon pyrosequencing was performed by the Research and Testing Laboratory (Lubbock, Texas, USA) as previously described [60]. Amplicons originating from the V1 to V3 regions were sequenced. Retrieved sequences were analyzed using MOTHUR [61]. Suspected chimeras were detected using the MOTHUR chimera.check module (∼10% of the sequences) and eliminated from further analysis, along with fragments shorter than 300 bp. Sequences were then aligned using the Silva-compatible alignment database [61] and a distance matrix was calculated. A 97% sequence-similarity threshold was used to group the sequences into operational taxonomic units (OTUs). Representatives of each OTU were classified with the MOTHUR classify.seqs module, and affiliation, down to the genus level, was verified by NCBI Blast analysis. Sequences were deposited in the NCBI SRA database under accession number SRA026104 and are available at http://departments.agri.huji.ac.il/plantpath/hadar-files/.

### Real-time Quantitative PCR (qPCR)

For each of the samples, four different qPCR assays targeting the 16S rRNA gene were done all: 1) total bacteria; 2) plant plastid; 4) *Massilia* spp.; 5) *Agrobacterium tumefaciens/Rhizobium radiobacter* clade (*Agrobacterium* spp.). The plant *tef* gene, coding for translation elongation factor 1, was also quantified and used for normalization of the results, as previously described [62]. All qPCR assays were conducted in polypropylene 96-well plates in a Mx3000P® QPCR System (Stratagene, California, USA). Each run (3 plates per assay) included a set of plasmid standards, with 8 orders of magnitude range. The standards and each sample within each treatment (72 samples in total) were tested in triplicates.

The assays for total bacteria, plant plastid and plant *tef* gene were performed as previously described [31]. The assay for *Massilia* spp. was modified from [31]: the reverse primer targeted to *Massilia* was modified and its sequence was 5′-TCHAGCCTTGCAGTCTCCARC-3′. This modification was made in order to increase the coverage of the *Massilia* group using the primer pair, following *in silico* examination of the ARB-Silva and RDP databases. The diversity of *Massilia* with *in silico* compatibility to the primer pair included all sequences related (97%–100% sequence similarity) to representative sequences of the 225 *Massilia* OTUs (of ≥20 sequences, *i.e*., 94% of all *Massilia* sequences in the entire dataset) retrieved by general bacterial pyrosequencing of 16S rRNA gene. PCR-amplified products were examined by gel electrophoresis to confirm the specificity of the amplification, and products were cloned by using a TOPO TA cloning kit. Plasmids were isolated by using a DNA-spin plasmid DNA extraction kit (iNtRON Biotechnology, Inc., Kyungki-Do, Korea). Ten randomly selected cloned inserts were sequenced in order to assert their identity. Preparation of a plasmid standard and reaction calibration was done as previously described [31]. The slope of the standard curve, correlation coefficient and amplification efficacy were calculated using MxPro™ QPCR Software analysis tools (Stratagene, California, USA) to be: −3.286, 0.971 and 101.5%, respectively.

An assay to quantify a group of *Agrobacterium tumefaciens/Rhizobium radiobacter* clade (*Agrobacterium* spp.) was developed. A 16S rRNA gene-targeting primer pair was designed using the ARB package [63], giving a group-specific 465-bp product. Primer sequences were: *A/R*144f: 5'-GCGGAATAGCTCCGGGAAACTGG-3' and *A/R*589r: 5'-CACCCCTGACTTAAATAT CCGC-3'. According to *in silico* analysis using RDP database, the primer pair targeted 18% of the genus *Rhizobium*, which included 68.35% of the sequences defined as *Rhizobium radiobacter* and 50.62% of sequences defined as *Agrobacterium* spp. Assessment of the assay specificity was done as described above for *Massilia*. Calibration of the real-time PCR reaction for this primer pair, including generation of a plasmid standard was done as previously described [31]. The slope of the standard curve, correlation coefficient and amplification efficacy were calculated using MxPro™ QPCR Software analysis tools (Stratagene, California, USA) to be: −3.258, 0.989 and 102.7%, respectively.

Reaction conditions were as follows: each 20 µl reaction contained: 10 µl Absolute Blue SYBR Green ROX mix (Thermo Fisher Scientific, Surrey, UK), 1.25 µl of each primer (10 µM), 6.5 µl H_2_O, and 1 µl template DNA. PCR conditions were: 15 min at 95°C, followed by 40 cycles of 95°C for 30 s, 60°C (or 58°C for the total bacteria primer pair) for 30 s, and 72°C for 30 s. Melting-curve analysis of the PCR products was conducted following each assay to confirm PCR specificity.

qPCR data were analyzed with MxPro™ QPCR Software analysis tools. Additional statistical analyses were performed using STATISTICA® (version 7.1) software (Stat Soft Inc., Oklahoma, USA) and MATLAB. One way Analysis of Variance (ANOVA), followed by post-hoc Tukey test (*P*<0.05) was used to compare the absolute abundances of *Massilia* in samples of different treatments sampled following 1 day of germination (including *Pythium* inoculated samples). Factorial ANOVA was used for comparing the absolute abundance of *Massilia* spp. or *Agrobacterium* spp. between the treatments and different plant growth stages (*P*<0.05) on including the *Pythium* inoculation treatment sampled only at the germination stage. This was followed by a post-hoc Newman-Keuls test for determination of the critical range for *P*<0.05.

Correspondence between relative abundance and absolute abundance values were examined for both *Massilia* spp. and *Agrobacterium* spp. populations. Based on the data distribution, non-linear regression models were considered. Non-linear robust regression analysis was performed using the Tukey bi-square weights method (MATLAB Curve Fitting Toolbox, MathWorks). This method reduces the impact of outliers in the data by differential weighing [64]. The model parameters estimates were iteratively determined using the Levenberg-Marquadt optimization method. A two-parameter model [Y = a·exp^(b·X)^+c] and a three parameter model [Y = a·exp^(b·X)^+c] were evaluated. For examination of the models goodness of fit, the coefficient of determination (R-square), root mean squared error (RMSE), were calculated and compared, and the Wald–Wolfowitz Runs Test of randomness were performed [64]–[66]. For the Runs Test of randomness the test statistic Z value was calculated according to the equation: Z = [R-E_(R)_]/V_(R)_^½^, where R is the number of runs, E_(R)_ = [2pn/(p+n)]+1 and V_(R)_ = [2pn(2pn–p–n)]/[(p+n)^2^(p+n–1)], where p and n are the number of residuals with positive values or negative values respectively. The associated two-tailed probability of Z was then calculated.

### Fluorescence *in situ* Hybridization (FISH) and Confocal Laser Scanning Microscopy (CLSM)

An oligonucleotide probe targeting the *Massilia* 16S rRNA gene was prepared using the sequence of primer Oxal_656R described above and was Cy3-labeled at the 5′ end. Calibration and specificity examination of the probe were performed using a *Massilia-*related soil isolate [67] and several non-target bacterial strains, including *Escherichia coli*, *Pseudomonas putida*, *Agrobacterium tumefaciens*, *Streptomyces* sp., *Sphingomonas* sp., *Flavobacterium* sp. and *Bacillus subtilis*. Hybridization stringency was optimized by adjustment of hybridization buffer formamide concentrations (20% to 50% v/v in steps of 5%) and accordingly adjustment of sodium chloride concentration in the wash buffer. In addition, different incubation periods were examined (from 2 to 16 hours). Hybridization was found to be optimal for this probe using hybridization buffer adjusted to 30%–35% formamide with 2 to 6 hours of hybridization at 46°C.

FAA-fixed seed coat, radicle or root samples were washed twice with phosphate buffered saline (PBS) and then dehydrated by soaking in increasing concentrations of ethanol (10 min at each of the following concentrations: 50%, 70%, 85%, 100%). Samples were left to dry for 10 min, then pre-warmed (46°C) hybridization buffer (20 mM Tris-HCl pH 8.0, 0.9 M NaCl, 35% formamide, 0.01% sodium dodecyl sulfate [SDS]) was added. An equimolar mixture of Cy5-labeled probes EUB338 [68], EUB338II and EUB338III [69] and the Cy3-labeled *Massilia-*targeting probe were added to a final concentration of 2.5 ng µL^−1^ for each probe. Samples were then incubated at 46°C for 2 h, washed with pre-warmed wash buffer (20 mM Tris-HCl pH 8.0, 80 mM NaCl, 50 mM EDTA, 0.01% SDS) for 15 min at 48°C and washed twice in PBS. 4',6-diamidino-2-phenylindole (DAPI) staining was performed with 0.2 mg mL^−1^ DAPI in PBS under ambient conditions for 5 min. Samples were then mounted on glass slides and viewed under an IX81Olympus FluoView 500 confocal microscope (Olympus, Tokyo, Japan). Detection specificity was confirmed using controls consisting of samples of the different plant tissues with no probe, as well as samples with Cy5-labeled probe complementary to the EUB338 probe.

### Effect of Organic Amendment on Relative Abundance of *Massilia* spp. and *Agrobacterium* spp. in Soil

Samples were kindly provided by Eyal Klein and Prof. Abraham Gamliel, Institute of Agricultural Engineering, Agricultural Research Organization of Israel. The experimental setup was as described by Klein *et al*., 2011. Briefly, a pot experiment was conducted under greenhouse conditions (24–26°C, ambient light conditions). For this experiment, a sandy soil from Rehovot was used (94% sand, 2% silt, 4% clay, 0.12% organic matter, pH 7.9), is typical of many agricultural soils in Israel [70]. In addition, the soil was mixed with organic residues (air dried and ground leaves and stems) of wild rocket (*Diplotaxis tenuifolia* L.) at the rate of 1% w/w. This soil amendment resulted in persistent soil suppressiveness (at least 34 months) towards fungal plant pathogens [32]. Cucumber seeds were germinated and grown for 6 days in Rehovot soil, and then transplanted into 450 mL pots containing either soil or wild rocket amended soil. Five replicates were prepared for each soil type, 7 seedlings transplanted in each. For each replicate, the roots of the 7 seedlings were pooled 3 and 6 days after transplantation and treated together. Roots were separated from the shoots, washed twice in sterile saline solution (0.85% NaCl), sheared with sterilized scissors and mixed. DNA was extracted from 100 mg root samples using PowerSoil® DNA isolation kit (MO BIO Laboratories, Inc., Ca, USA) and used as template for real-time quantitative PCR of total bacteria and *Massilia*, as described above. The relative abundances were of *Massilia* spp. and of *Agrobacterium* spp. were compared between treatments and plant developmental stage by factorial ANOVA using the STATISTICA® (version 7.1) software, followed by post-hoc Tukey HSD test (*P*<0.05).

## Supporting Information

Table S1 **Numbers of 16S rRNA gene fragments sequences retrieved by high-throughput sequencing.**
(DOCX)
