# Supplementary material for: Ecology of Root Colonizing Massilia (Oxalobacteraceae)
Source: PLoS One. 2012 Jul 11;7(7):e40117. doi: 10.1371/journal.pone.0040117 (PMC3394795; doi:10.1371/journal.pone.0040117)
Supplement: Table S1 — Numbers of 16S rRNA gene fragments sequences retrieved by high-throughput sequencing. (DOCX) [file pone.0040117.s001.docx]

Table S1: Numbers of 16S rRNA gene fragments sequences retrieved by high-throughput sequencing.

| Treatment | Day | Rep^1^ | Total | Chloroplast | Bacteria | Oxalobacteraceae |
| --- | --- | --- | --- | --- | --- | --- |
| Perlite | 1 | 2 | 11,704 | 11,653 | 51 | 1 |
|  | 2 | 4 | 34,782 | 3,484 | 31,298 | 13,953 |
|  | 7 | 3 | 59,086 | 3,928 | 55,158 | 34,034 |
|  | 21 | 3 | 46,184 | 39,190 | 6,994 | 216 |
| Perlite+Compost | 1 | 3 | 14,912 | 128 | 14,784 | 3,011 |
|  | 2 | 4 | 15,657 | 903 | 14,754 | 2,113 |
|  | 7 | 3 | 30,452 | 10,088 | 20,364 | 321 |
|  | 21 | 2 | 14,104 | 12,153 | 1,951 | 8 |
| Perlite+*Pythium* | 1 | 3 | 23,204 | 372 | 22,832 | 15,228 |
| Perlite+Compost+*Pyhtium* | 1 | 3 | 13,292 | 315 | 12,977 | 4,182 |

^1^ Number of replicates sequenced.
